# Alkaloid Constituents of *Ficus hispida* and Their Antiinflammatory Activity

**DOI:** 10.1007/s13659-020-00233-5

**Published:** 2020-02-18

**Authors:** Xin-Yu Jia, Yong-Mei Wu, Jing-Ya Li, Chun Lei, Ai-Jun Hou

**Affiliations:** 1 School of Pharmacy, State Key Laboratory of Medical Neurobiology, Fudan University, Shanghai, 201203 China; 2 National Center for Drug Screening, Shanghai Institute of Materia Medica, Chinese Academy of Sciences, Shanghai, 201203 China

**Keywords:** *Ficus hispida*, Moraceae, Alkaloids, Antiinflammation, Nuclear factor-*κB*

## Abstract

Four new alkaloids, ficuhismines A–D (**1**–**4**), together with three known ones, were isolated from *Ficus hispida*. Their structures were elucidated by spectroscopic analysis and chemical method. The new compounds represent the first amine alkaloids with a rhamnosyl moiety (**1**–**2**) or with a *N*-oxide motif (**2**–**4**) from the genus *Ficus*. Compound **2** showed potent inhibitory effect in nuclear factor-*κ*B (NF-*κ*B) pathway luciferase assay with IC_50_ value of 0.52 ± 0.11 μM.

**Electronic supplementary material:**

The online version of this article (10.1007/s13659-020-00233-5) contains supplementary material, which is available to authorized users.

## Introduction

The genus *Ficus* (Moraceae) comprises approximately 1000 species all over the world. They mainly distribute in tropical and subtropical areas and show diversity particularly in Southeast Asia [1]. In addition to the well-known fig (“Wu Hua Guo”) and banyan trees, many *Ficus* plants possess important medicinal values. The plant *Ficus hispida* L.f. is a herbal medicine that has been used in China and India as a remedy for bronchitis, dysentery, rheumatism, and skin disorders [2, 3]. The extracts from its different parts have been reported to show antiinflammatory, antidiabetic, antitumor, and hepatoprotective activities [4–7]. Primarily known for the rich flavonoids and triterpenoids [8], alkaloids with antitumor and vasorelaxant activities have also been discovered from *F. hispida* [9, 10]. Some alkaloids and *α*-glucosidase inhibitory flavonoids from this plant have been reported previously by our group [11].

**Fig. 1:**
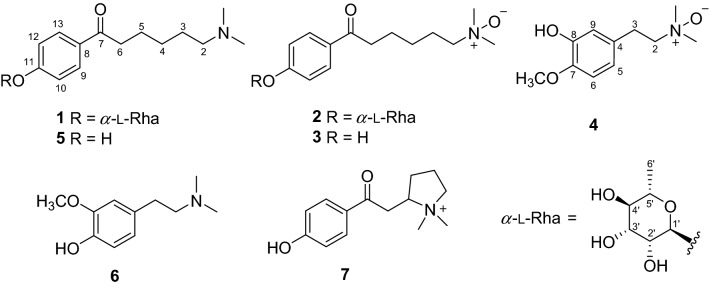
Structures of compounds **1**–**7**

Nuclear factor-*κ*B (NF-*κ*B) is a key regulator of inflammation. Activation of NF-*κ*B initiates inflammation-associated metabolic disease, such as obesity, type 2 diabetes, and atherosclerosis [12]. In order to discover antiinflammatory natural products against metabolic diseases, alkaloid constituents of *F. hispida* were reinvestigated, and their inhibitory effects in NF-*κ*B pathway luciferase assay were evaluated. Seven alkaloids including four new ones (ficuhismines A–D, **1**–**4**) were isolated from the twigs and leaves of *F. hispida* (Fig. 1). The new compounds represent the first amine alkaloids with a rhamnosyl moiety (**1**–**2)** or with a *N*-oxide motif (**2**–**4**) from the genus *Ficus*. Compound **2** showed potent NF-*κ*B inhibitory activity. In this paper, the structural identification and bioactivity evaluation of the alkaloids are discussed.

## Results and Discussion

**Fig. 2:**
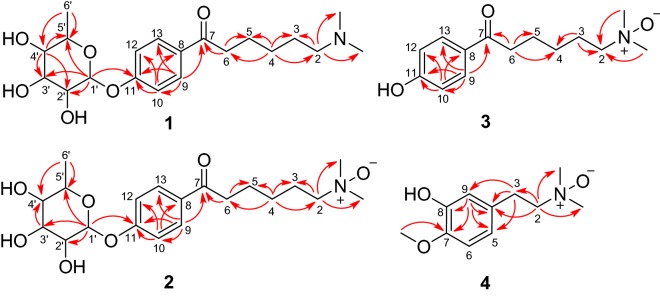
Key HMBC (H → C) correlations of compounds **1**–**4**

Compound **1** was assigned the molecular formula C_20_H_31_NO_6_ by the (+)-HRESIMS ion at *m*/*z* 382.2232 [M+H]^+^ (calcd for C_20_H_32_NO_6_, 382.2224). The IR spectrum of **1** showed absorption bands for OH (3385 cm^−1^), carbonyl (1675 cm^−1^), and aromatic (1599 cm^−1^) functionalities. The 1D NMR (Table 1) and HSQC spectra displayed signals of a carbonyl group (*δ*_C_ 201.0, C-7), a *para*-substituted benzene ring (*δ*_H_ 7.96, H-9/H-13; 7.13, H-10/H-12; *δ*_C_ 132.3, C-8; 131.4, C-9/C-13; 117.1, C-10/C-12; 161.8, C-11), a rhamnopyranosyl moiety (*δ*_H_ 5.52, H-1′; 3.98, H-2′; 3.81, H-3′; 3.44, H-4′; 3.54, H-5′; 1.18, H_3_-6′; *δ*_C_ 99.6, C-1′; 71.8, C-2′; 72.1, C-3′; 73.7, C-4′; 71.0, C-5′; 18.0, C-6′), a dimethylamino moiety (*δ*_H_ 2.85; *δ*_C_ 43.4), and five methylenes. The anomeric configuration of the l-rhamnopyranosyl moiety was determined as *α* by the chemical shifts of C-3′ and C-5′ [13]. The aforementioned information indicated **1** was an amine alkaloid rhamnoside. In the HMBC spectrum (Fig. 2), the correlations of H_2_-2/Me_2_N, C-3; H_2_-4/C-2, C-3, C-5, C-6; H_2_-6/C-5, C-7; H-9/C-7, C-10, C-11, C-13; and H-10/C-8, C-11, C-12 constructed the aglycone motif as a 6-(dimethylamino)-1-(4-hydroxyphenyl)-1-hexanone. The H-1′/C-11 HMBC correlation indicated that the sugar was connected to C-11. Acid hydrolysis of **1** afforded the aglycone and sugar units. The sugar was verified as l-rhamnose by TLC analysis and comparing its specific rotation ([α]_D_^25^ =  + 6.0) with that of the authentic l-rhamnose ([α]_D_^25^ =  + 8.0). Thus, the structure of **1** was elucidated as depicted, and the compound was given the name ficuhismine A.

**Table 1:** ^1^H (400 MHz) and ^13^C (150 MHz) NMR data for compounds **1**–**4** (in methanol-*d*_4_)

Pos.	**1**		**2**		**3**		**4**	
	*δ*_H_ (*J* in Hz)	*δ*_C_	*δ*_H_ (*J* in Hz)	*δ*_C_	*δ*_H_ (*J* in Hz)	*δ*_C_	*δ*_H_ (*J* in Hz)	*δ*_C_
2H	3.10 (m)	58.9	3.63 (m)	70.5	3.61 (m)	70.5	3.43 (m)	73.0
3	1.72 (m)	25.5	1.92 (m)	23.9	1.90 (m)	23.9	3.04 (m)	30.4
4	1.43 (m)	27.0	1.47 (m)	26.7	1.45 (m)	26.8		129.1
5	1.74 (m)	24.8	1.78 (m)	24.8	1.76 (m)	25.0	6.67 (br d, 8.0)	122.5
6	3.01 (t, 6.8)	38.6	3.04 (t, 7.2)	38.6	2.99 (t, 7.2)	38.4	6.70 (d, 8.0)	116.7
7		201.0		200.9		201.0		149.5
8		132.3		132.3		130.0		147.3
9	7.96 (d, 8.8)	131.4	7.97 (d, 8.8)	131.4	7.87 (d, 8.8)	131.8	6.85 (br s)	113.6
10	7.13 (d, 8.8)	117.1	7.15 (d, 8.8)	117.1	6.82 (d, 8.8)	116.3		
11		161.8		161.8		163.9		
12	7.13 (d, 8.8)	117.1	7.15 (d, 8.8)	117.1	6.82 (d, 8.8)	116.3		
13	7.96 (d, 8.8)	131.4	7.97 (d, 8.8)	131.4	7.87 (d, 8.8)	131.8		
1′	5.52 (br s)	99.6	5.53 (br s)	99.6				
2′	3.98 (br s)	71.8	4.00 (br s)	71.8				
3′	3.81 (dd, 9.6, 3.2)	72.1	3.82 (dd, 9.3, 3.3)	72.2				
4′	3.44 (t, 9.6)	73.7	3.46 (overlap)	73.7				
5′	3.54 (m)	71.0	3.55 (m)	71.0				
6′	1.18 (d, 6.0)	18.0	1.20 (d, 6.0)	18.0				
Me_2_N	2.85 (s)	43.4	3.46 (s)	56.4	3.45 (s)	56.4	3.17 (s)	58.6
MeO							3.82 (s)	56.4

Compound **2** was assigned the molecular formula C_20_H_31_NO_7_ by the (+)-HRESIMS (*m*/*z* 398.2165 [M+H]^+^; calcd for C_20_H_32_NO_7_, 398.2173), indicating six indices of hydrogen deficiency. Analysis of its NMR data (Table 1) implied that **2** possessed the same *α*-rhamnopyranosyl and *para*-substituted benzene moieties as **1**. The only difference was the 6-(dimethylamino)-1-hexanone side chain. In the ^13^C NMR spectrum of **2**, the obviously deshielded carbon signals of the dimethylamino moiety (Δ*δ*_C_ + 13.0) and the methylene at C-2 (Δ*δ*_C_ + 11.6) were observed. In consideration the indices of hydrogen deficiency and an additional oxygen atom in the molecular formula of **2**, the presence of a *N*-oxide motif was inferred. The HMBC data shown in Fig. 2 confirmed the structure of **2**. The sugar was identified as l-rhamnose by the same protocol as that of **1**. Compound **2** was thus elucidated as an *N*-oxide of **2** and named ficuhismine B.

Compound **3** was assigned the molecular formula C_14_H_21_NO_3_ by the (+)-HRESIMS ion at *m*/*z* 252.1595 [M+H]^+^ (calcd for C_14_H_22_NO_3_, 252.1594). Its 1D (Table 1) and 2D NMR data (Fig. 2) indicated that **3** was an aglycone of **2**. The structure of **3** was thus elucidated, and the compound was named ficuhismine C.

Compound **4** was assigned the molecular formula C_11_H_17_NO_3_ by the (+)-HRESIMS ion at *m*/*z* 212.1281 [M+H]^+^ (calcd for C_11_H_18_NO_3_, 212.1281). The ^1^H and ^13^C NMR spectra of **4** (Table 1) revealed signals for a dimethylamino moiety, two methylenes, a methoxy group, and a 1, 2, 5-trisubstituted benzene motif. Similar to **2** and **3**, the downfield-shifted ^13^C resonances of the dimethylamino moiety and the methylene at C-2 proved that **4** was also an amino alkaloid *N*-oxide. The HMBC correlations of H_2_-2/Me_2_N, C-3, C-4; H_2_-3/C-4, C-5, C-9; H-9/C-4, C-5, C-7, C-8; and MeO/C-7 (Fig. 2) confirmed the structure of **4** as depicted. The structure of **4** was thus elucidated, and the compound was named ficuhismine D.

The known compounds were identified as ficushispimine C (**5**) [11] magnosprengerine (**6**) [14], and ficushispimine A (**7**) [11] (Fig. 1) by comparison of their spectroscopic data with those reported.

All the isolated alkaloids were tested in vitro for their antiinflammatory effects. Ficuhismine B (**2**) exhibited significant inhibitory activity in NF-*κ*B pathway luciferase assay with IC_50_ value of 0.52 ± 0.11 μM. Bortezomib (PS-341) was used as the positive control (IC_50_ = 0.12 ± 0.04 μM) in this test. All the other compounds were inactive. It seemed that both l-rhamnosyl and *N*-oxide moieties are necessary for the NF-*κ*B inhibition.

Alkaloids are one of the main bioactive ingredients of *F. hispida*. Amphetamine [9, 11], piperidine [9], pyrrolidine [10, 11], and isoquinoline [15] alkaloids have been reported from this plant previously. In this research, amine alkaloids with a rhamnosyl group (**1**–**2**) or with a *N*-oxide moiety (**2**–**4**) were discovered for the first time from the genus *Ficus*. Compound **2** with both rhamnosyl and *N*-oxide moieties showed potent NF-*κ*B inhibitory activity, which may provide useful information for mining or designing drug leads against inflammation-related metabolic diseases.

## Experimental Section

### General Experimental Procedures

Optical rotations were recorded on a Rudolph Autopol IV-T polarimeter. UV spectra were recorded on a Hitachi U-2900 UV–Vis spectrophotometer. IR spectra were recorded on a ThermoFisher Nicolet iS5 FT-IR spectrometer. NMR spectra were acquired on Varian Mercury Plus 400 instrument and Bruker Avance III HD 600 spectrometer using CD_3_OD (*δ*_H_ 3.31 and *δ*_C_ 49.0). HRESIMS were obtained on an AB SCIEX 5600+ Q-TOF mass spectrometer. MCI gel CHP-20P (75–150 μm, Mitsubishi Chemical Corporation, Tokyo, Japan), ODS gel (50 μm, YMC Co., Ltd., Japan), and Sephadex LH-20 gel (GE Healthcare Bio-Sciences, USA) were used for column chromatography. Precoated silica gel GF254 plates (Qingdao Haiyang Chemical Co., Ltd., China) were used for TLC analysis. Semi-preparative HPLC was performed on a Shimadzu Essentia LC-16 with a UV detector (210 and 254 nm) and a Kromasil C_18_ column (150 × 10 mm, 5 μm, AkzoNobel, Co., Sweden).

### Plant Material

The twigs and leaves of *Ficus hispida* were collected in Puer City, Yunnan Province, People’s Republic of China, in September 2015. The plant material was identified by Dr. Yun Kang, School of Pharmacy, Fudan University. A voucher specimen (TCM 2015-09-02 Hou) has been deposited at the Herbarium of the Department of Pharmacognosy, School of Pharmacy, Fudan University.

### Extraction and Isolation

The plant material (10.0 kg) was ground and percolated with 95% EtOH (30 L) at room temperature. The filtrate was evaporated under reduced pressure to produce a crude extract (1.5 kg). It was dissolved in tartaric acid solution (pH 2.0), basified using saturated sodium carbonate solution to pH 13, and then partitioned with CHCl_3_ to give a total alkaloid extract (1.2 g). This extract was subjected to MCI column chromatography (CC) (MeOH/H_2_O, from 1:9 to 10:0) to provide fractions A–E. Fractions A–D were separated over CC (ODS gel, MeOH/H_2_O, 10:90, 20:80, 30:70, 100:0) to give fractions A1–A4, B1–B4, C1–C4, and D1–D4, respectively. Fr. A1 was loaded on a Sephadex LH-20 column (CH_2_Cl_2_/MeOH, 1:1) and then purified by semi-preparative HPLC (MeOH/H_2_O, 9:91, flow rate 2 mL/min) to afford **3** (2.3 mg, *t*_R_ 16.5 min). Fractions A2 and B1 were purified by semi-preparative HPLC to give **5** (MeCN/H_2_O, 16:84, flow rate 2 mL/min, 2.3 mg, *t*_R_ 23 min) and **7** (MeCN/H_2_O, 6:94, flow rate 2 mL/min, 2.5 mg, *t*_R_ 15 min), respectively. Compounds **4** (MeOH/H_2_O, 3:97, flow rate 4 mL/min, 5.0 mg, *t*_R_ 31 min) and **6** (MeCN/H_2_O, 10:90, flow rate 2 mL/min, 1.5 mg, *t*_R_ 26 min) were isolated by semi-preparative HPLC from fractions C1 and C2, respectively. Compounds **2** (MeOH/H_2_O, 3:97, flow rate 4 mL/min, 2.0 mg, *t*_R_ 31 min) and **1** (MeCN/H_2_O, 11:89, flow rate 4 mL/min, 2.0 mg, *t*_R_16.0 min) were purified by semi-preparative HPLC from fractions D1 and D3, respectively.

### Spectroscopic Data of Compounds

#### Ficuhismine A (1)

White solid; [*α*]_D_^25^ = − 61.0 (*c* 0.1, MeOH); UV (MeOH) *λ*_max_ (log *ε*) 206 (4.28), 262 (4.32) nm; IR (KBr) *ν*_max_ 3385, 2915, 1675, 1599, 1383, 1181, 1141, 1075, 632 cm^−1^; ^1^H NMR and ^13^C NMR data: Table 1; HRESIMS *m/z* 382.2232 [M+H]^+^ (calcd for C_20_H_32_NO_6_, 382.2224).

#### Ficuhismine B (2)

White solid; [*α*]_D_^25^ = − 70.0 (*c* 0.1, MeOH); UV (MeOH) *λ*_max_ (log *ε*) 206 (4.32), 264 (4.41) nm; IR (KBr) *ν*_max_ 3372, 2935, 1675, 1600, 1421, 1196, 1179, 1140, 1073, 1014, 971, 835 cm^−1^; ^1^H NMR and ^13^C NMR data: Table 1; HRESIMS *m/z* 398.2165 [M+H]^+^ (calcd for C_20_H_32_NO_7_, 398.2173).

#### Ficuhismine C (3)

White solid; UV (MeOH) *λ*_max_ (log *ε*) 204 (3.87), 216 (3.90), 274 (4.03) nm; IR (KBr) *ν*_max_ 3412, 2914, 1674, 1604, 1193, 1178, 1142, 1073, 637 cm^−1^; ^1^H NMR and ^13^C NMR data: Table 1; HRESIMS *m/z* 252.1595 [M+H]^+^ (calcd for C_14_H_22_NO_3_, 252.1594).

#### Ficuhismine D (4)

White solid; UV (MeOH) *λ*_max_ (log *ε*) 204 (3.81), 216 (3.80), 274 (3.97) nm; IR (KBr) *ν*_max_ 3409, 2950, 2937, 2855, 2581, 1599, 1524, 1454, 1387, 1280, 1033 cm^−1^; ^1^H NMR and ^13^C NMR data: Table 1; HRESIMS *m/z* 212.1281 [M+H]^+^ (calcd for C_11_H_18_NO_3_, 212.1281).

### Acid Hydrolysis of Compounds 1 and 2

Compound **1** (1 mg) was added to a solution of H_2_O (3 mL), dioxane (3 mL), and HCl (0.5 mL), and then kept at 60 °C for 4 h. The reaction mixture was quenched with NaHCO_3_ and extracted with EtOAc. The aqueous phase was concentrated under reduced pressure. The residue was dissolved in H_2_O for TLC analysis with the authentic l-rhamnose (EtOAc/MeOH/AcOH/H_2_O, 10:3:3:2, v/v, R_f_: 0.6) and for optical rotation measurement {the aqueous phase: [α]_D_^25^ =  + 6.0 (*c* 0.1 H_2_O); l-rhamnose: [α]_D_^25^ =  + 8.0 (*c* 0.1 H_2_O)}. Compound **2** was operated in the same way as **1**.

### Luciferase Assay

HEK293/NF-*κ*B cells were prepared as reported [16]. The luciferase assay procedure was performed according to the reported methods [17]. The IC_50_ values were calculated using Graphpad prism 8.

## Electronic supplementary material

Below is the link to the electronic supplementary material.

Electronic supplementary material 1 (DOCX 6474 kb)
