# Supplementary material for: Alkaloid Constituents of Ficus hispida and Their Antiinflammatory Activity
Source: Nat Prod Bioprospect. 2020 Feb 18;10(1):45–9. doi: 10.1007/s13659-020-00233-5 (PMC7046851; doi:10.1007/s13659-020-00233-5)

**Supplementary data for**

**Alkaloid Constituents of *Ficus hispida* and Their Antiinflammatory Activity**

Xin-Yu Jia^1^, Yong-Mei Wu^2^, Jing-Ya Li^2^, Chun Lei^1*^, Ai-Jun Hou^1*^

^1^School of Pharmacy, State Key Laboratory of Medical Neurobiology, Fudan University, Shanghai 201203, China

^2^National Center for Drug Screening, Shanghai Institute of Materia Medica, Chinese Academy of Sciences, Shanghai 201203, China

Corresponding authors:

E-mails: chunlei@fudan.edu.cn (C. Lei); ajhou@shmu.edu.cn (A.-J. Hou)

**Table of Contents**

[Fig. S1. ^1^H NMR spectrum of compound **1** (in CD_3_OD, 400 MHz) 3](#_Toc29769273)

[Fig. S2. ^13^C NMR and DEPT spectra of compound **1** (in CD_3_OD, 150 MHz) 3](#_Toc29769274)

[Fig. S3. HSQC spectrum of compound **1** (in CD_3_OD, 600 MHz) 4](#_Toc29769275)

[Fig. S4. HMBC spectrum of compound **1** (in CD_3_OD, 600 MHz) 4](#_Toc29769276)

[Fig. S5. HRESIMS spectrum of compound **1** 5](#_Toc29769277)

[Fig. S6. ^1^H NMR spectrum of compound **2** (in CD_3_OD, 400 MHz) 5](#_Toc29769278)

[Fig. S7. ^13^C NMR and DEPT spectra of compound **2** (in CD_3_OD, 150 MHz) 6](#_Toc29769279)

[Fig. S8. HSQC spectrum of compound **2** (in CD_3_OD, 600 MHz) 6](#_Toc29769280)

[Fig. S9. HMBC spectrum of compound **2** (in CD_3_OD, 600 MHz) 7](#_Toc29769281)

[Fig. S10. HRESIMS spectrum of compound **2** 7](#_Toc29769282)

[Fig. S11. ^1^H NMR spectrum of compound **3** (in CD_3_OD, 400 MHz) 8](#_Toc29769283)

[Fig. S12. ^13^C NMR spectrum of compound **3** (in CD_3_OD, 150 MHz) 8](#_Toc29769284)

[Fig. S13. HSQC spectrum of compound **3** (in CD_3_OD, 600 MHz) 9](#_Toc29769285)

[Fig. S14. HMBC spectrum of compound **3** (in CD_3_OD, 600 MHz) 9](#_Toc29769286)

[Fig. S15. HRESIMS spectrum of compound **3** 10](#_Toc29769287)

[Fig. S16. ^1^H NMR spectrum of compound **4** (in CD_3_OD, 400 MHz) 10](#_Toc29769288)

[Fig. S17. ^13^C NMR and DEPT spectra of compound **4** (in CD_3_OD, 150 MHz) 11](#_Toc29769289)

[Fig. S18. HSQC spectrum of compound **4** (in CD_3_OD, 600 MHz) 11](#_Toc29769290)

[Fig. S19. HMBC spectrum of compound **4** (in CD_3_OD, 600 MHz) 12](#_Toc29769291)

[Fig. S20. HRESIMS spectrum of compound **4** 12](#_Toc29769292)

# Fig. S1. ^1^H NMR spectrum of compound 1 (in CD_3_OD, 400 MHz)


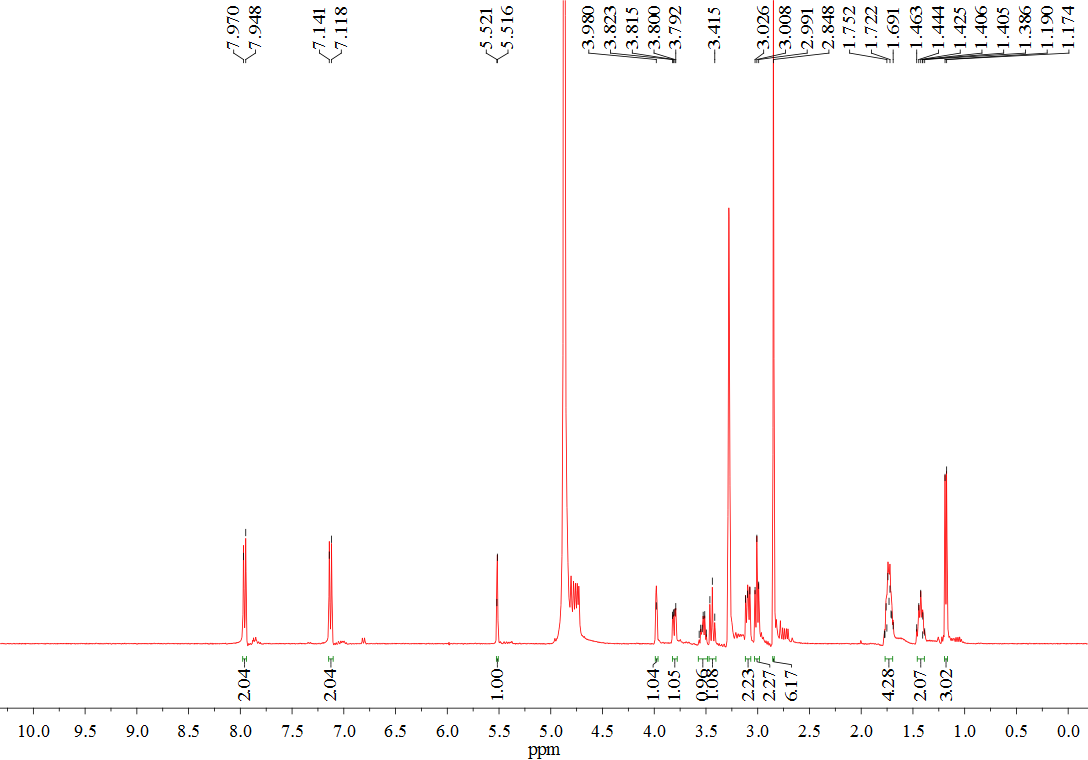


# Fig. S2. ^13^C NMR and DEPT spectra of compound 1 (in CD_3_OD, 150 MHz)


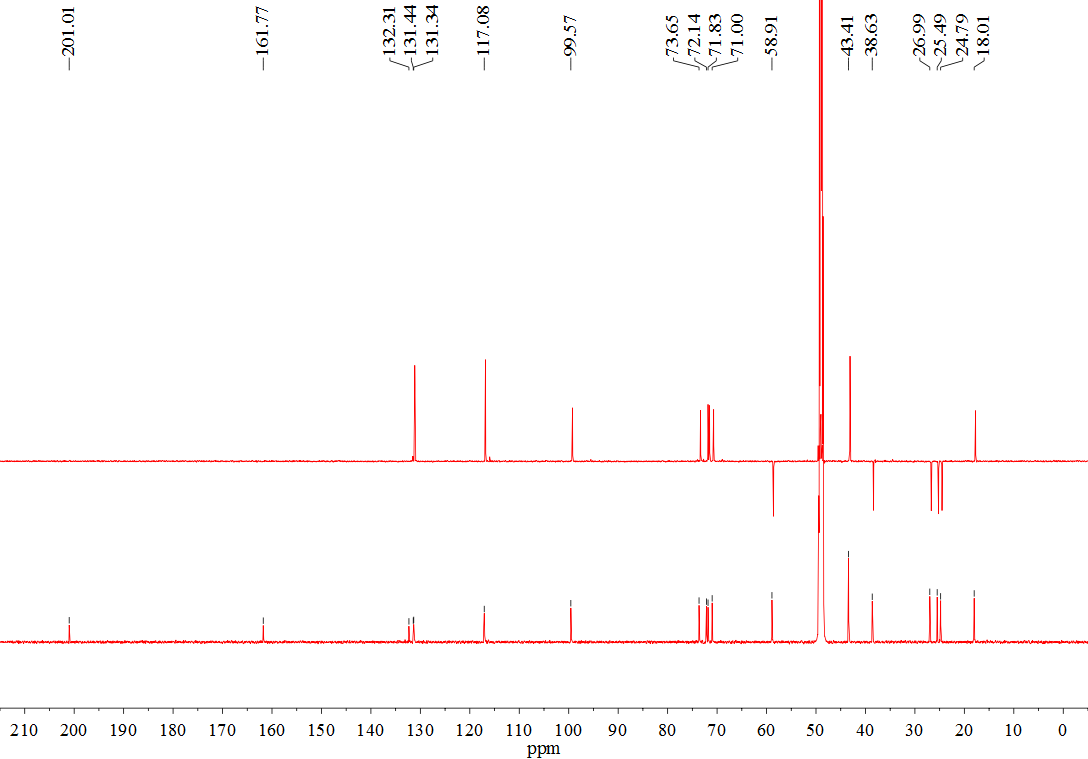


# Fig. S3. HSQC spectrum of compound 1 (in CD_3_OD, 600 MHz)


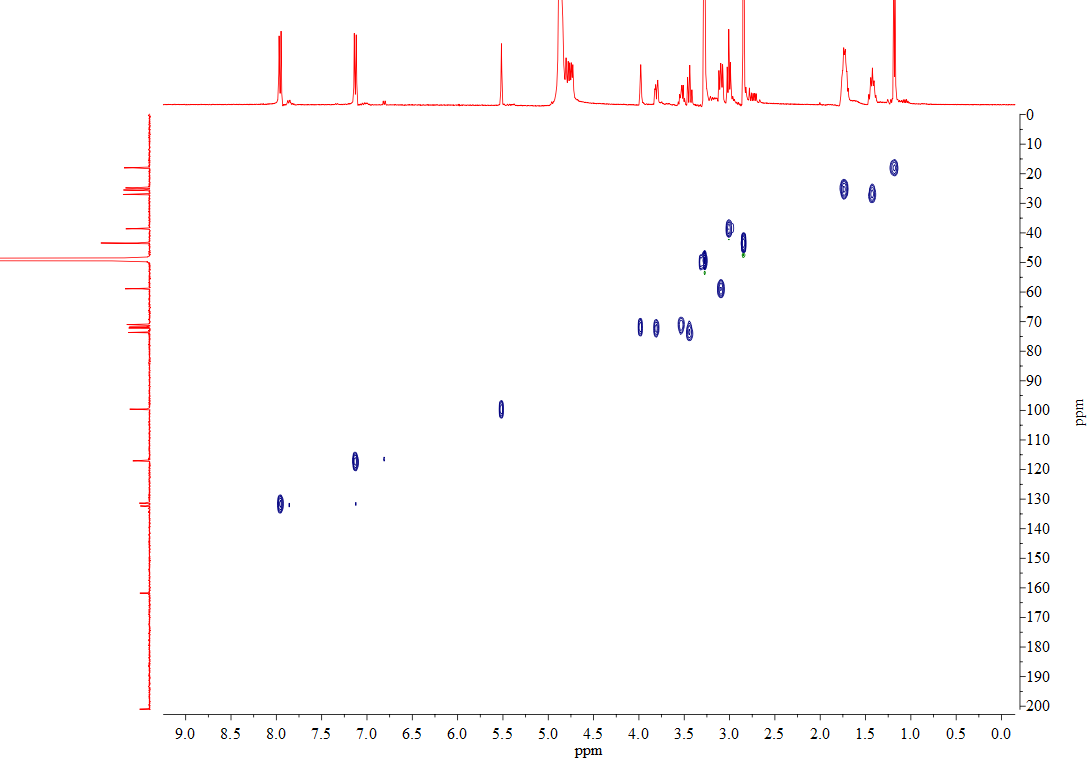


# Fig. S4. HMBC spectrum of compound 1 (in CD_3_OD, 600 MHz)


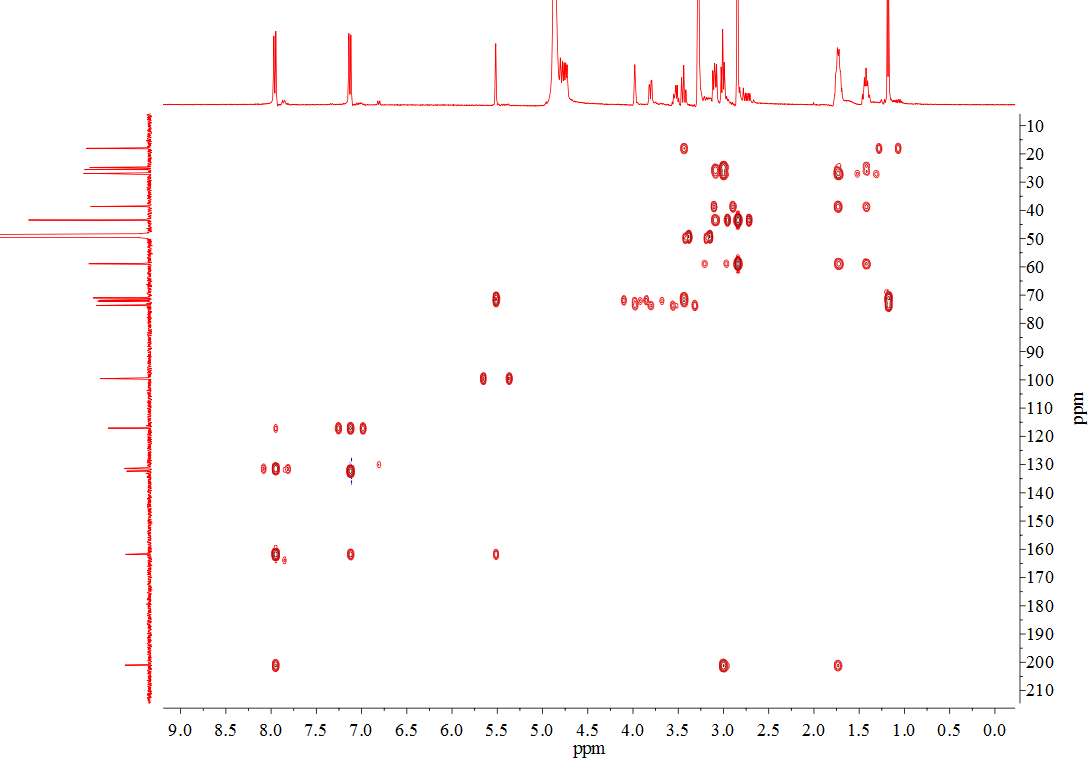


# Fig. S5. HRESIMS spectrum of compound 1


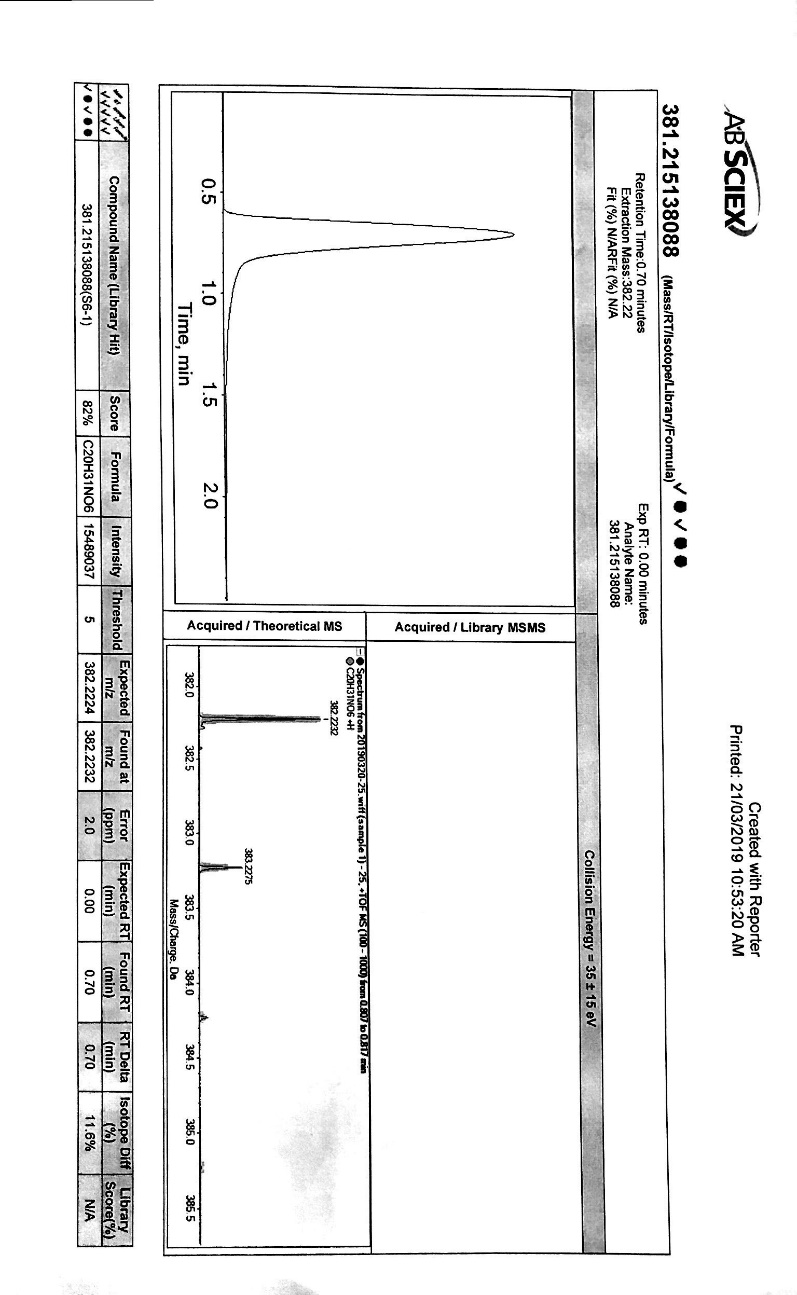


# Fig. S6. ^1^H NMR spectrum of compound 2 (in CD_3_OD, 400 MHz)


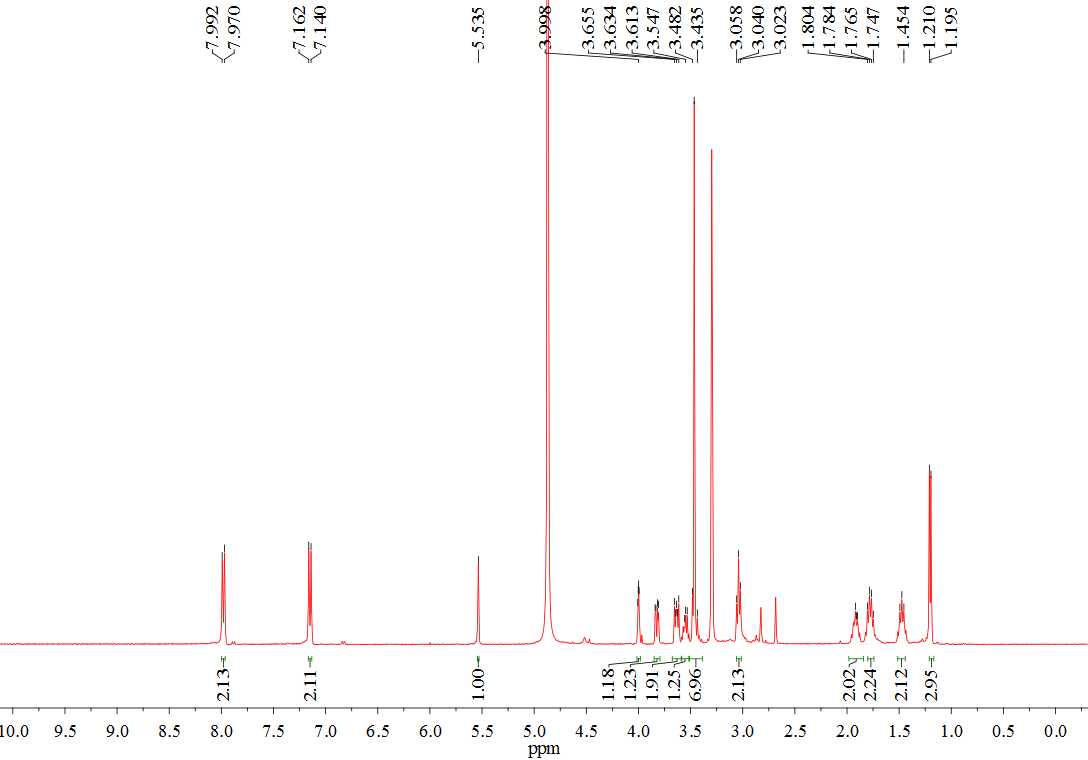


# Fig. S7. ^13^C NMR and DEPT spectra of compound 2 (in CD_3_OD, 150 MHz)


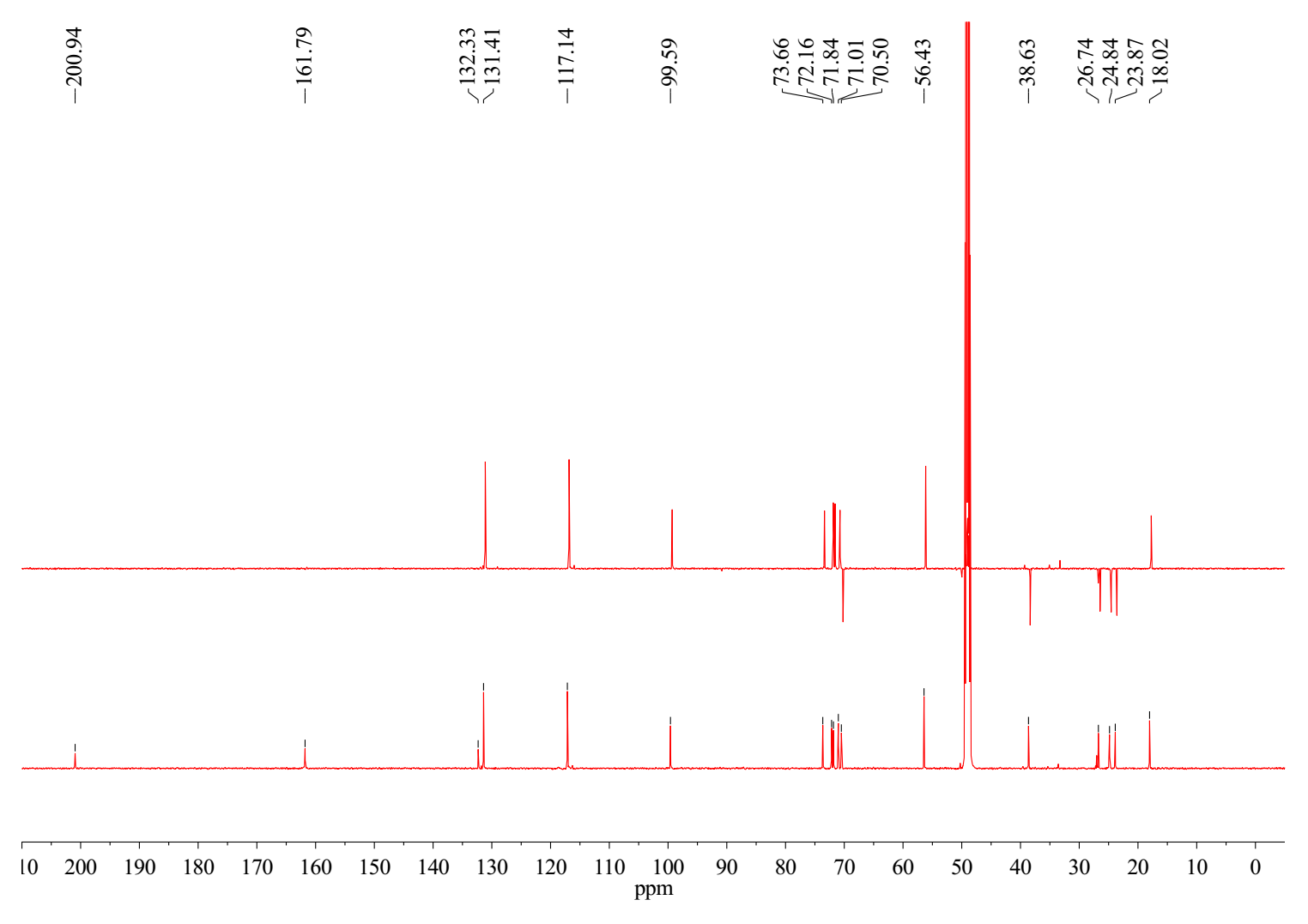


# Fig. S8. HSQC spectrum of compound 2 (in CD_3_OD, 600 MHz)


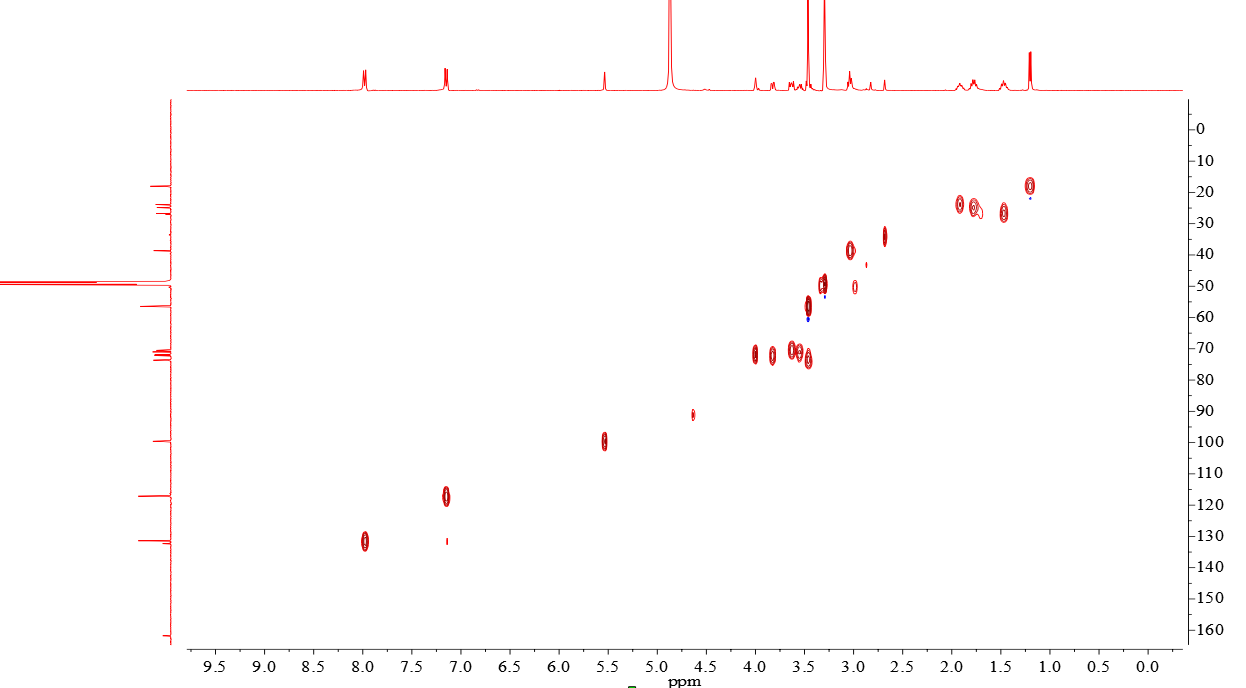


# Fig. S9. HMBC spectrum of compound 2 (in CD_3_OD, 600 MHz)


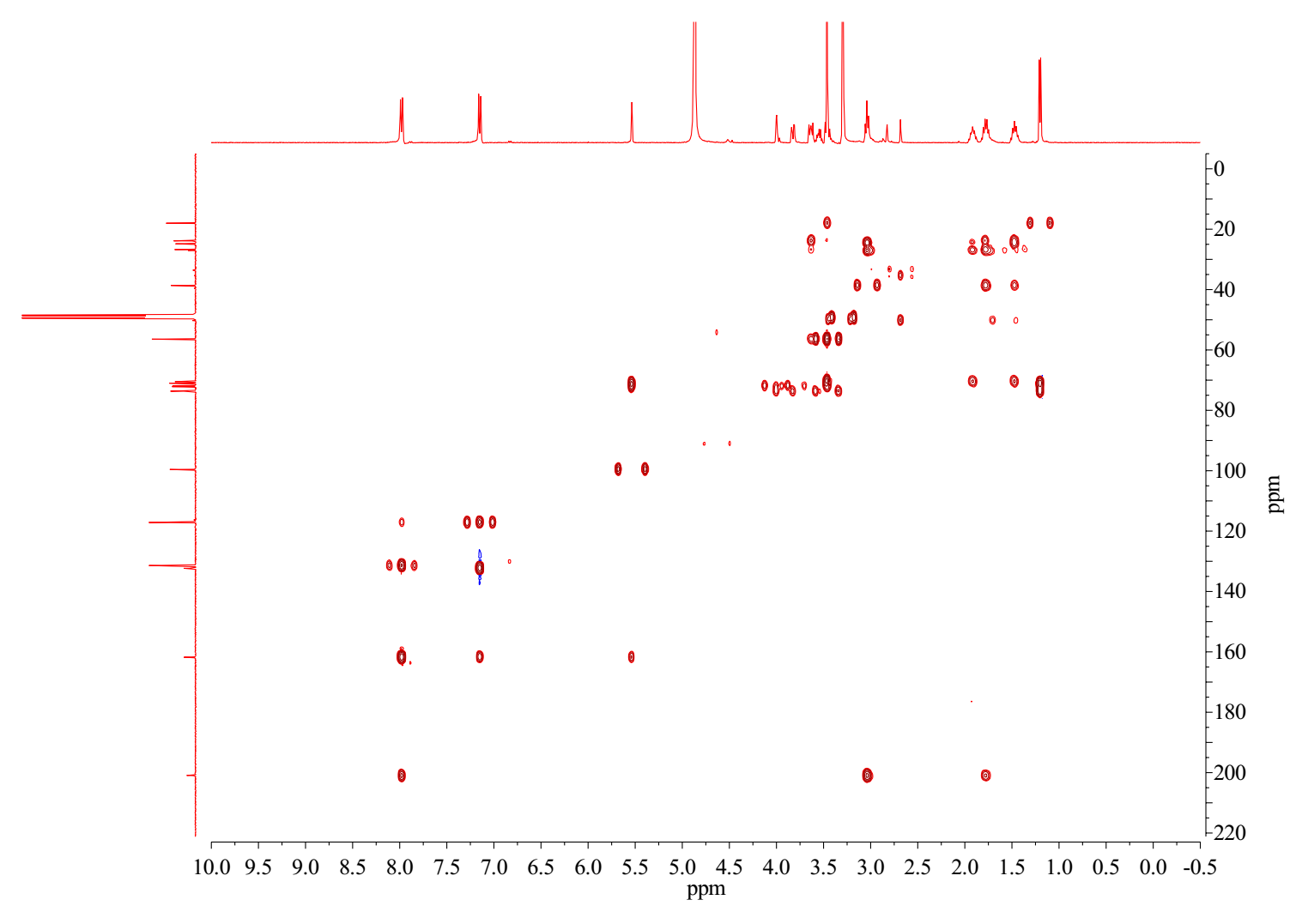


# Fig. S10. HRESIMS spectrum of compound 2


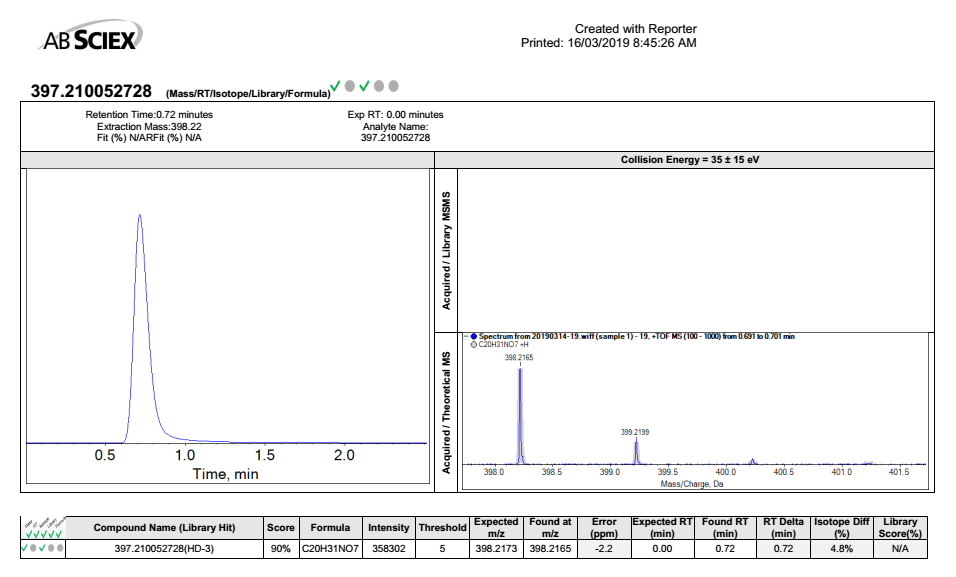


# Fig. S11. ^1^H NMR spectrum of compound 3 (in CD_3_OD, 400 MHz)


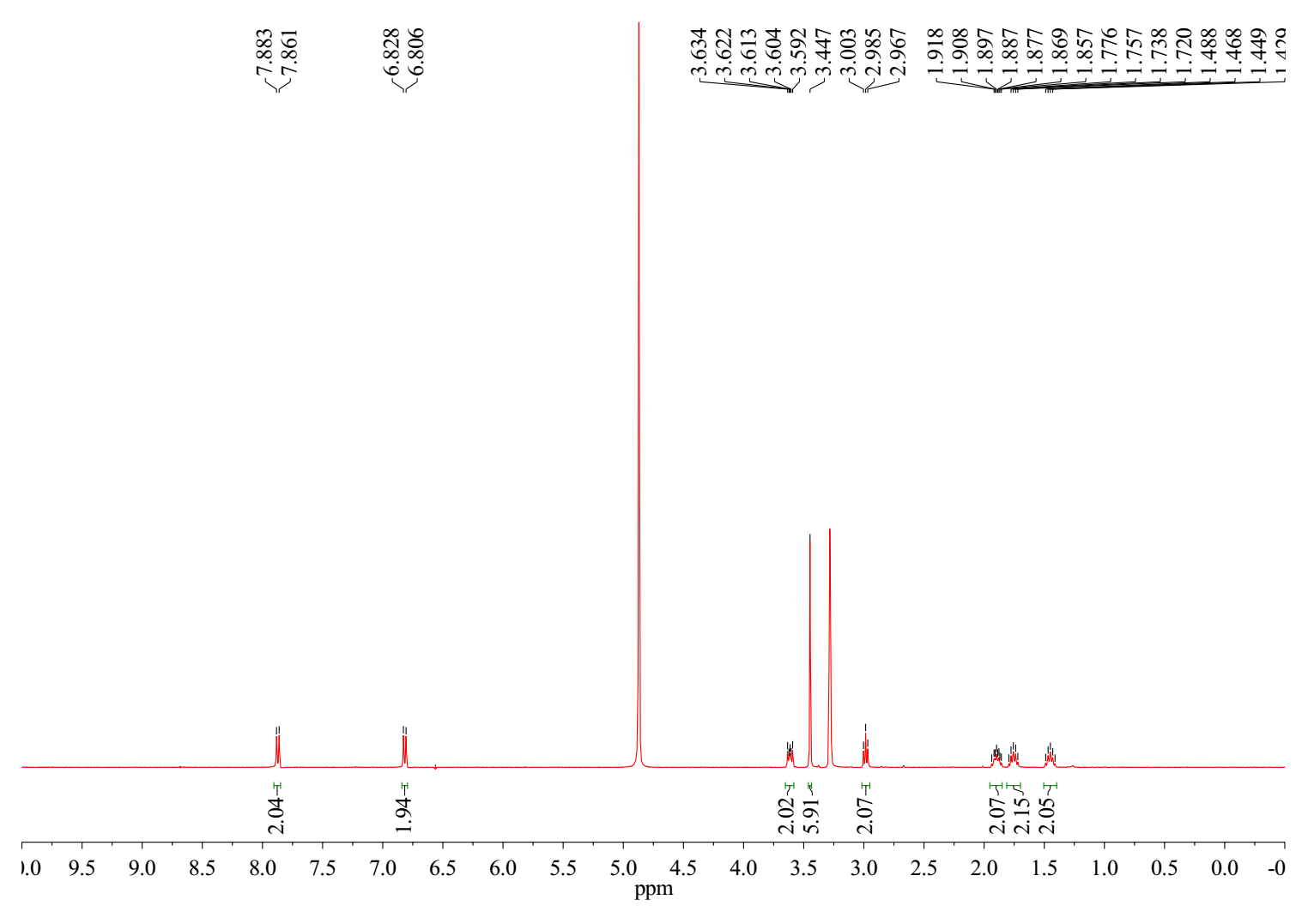


# Fig. S12. ^13^C NMR spectrum of compound 3 (in CD_3_OD, 150 MHz)


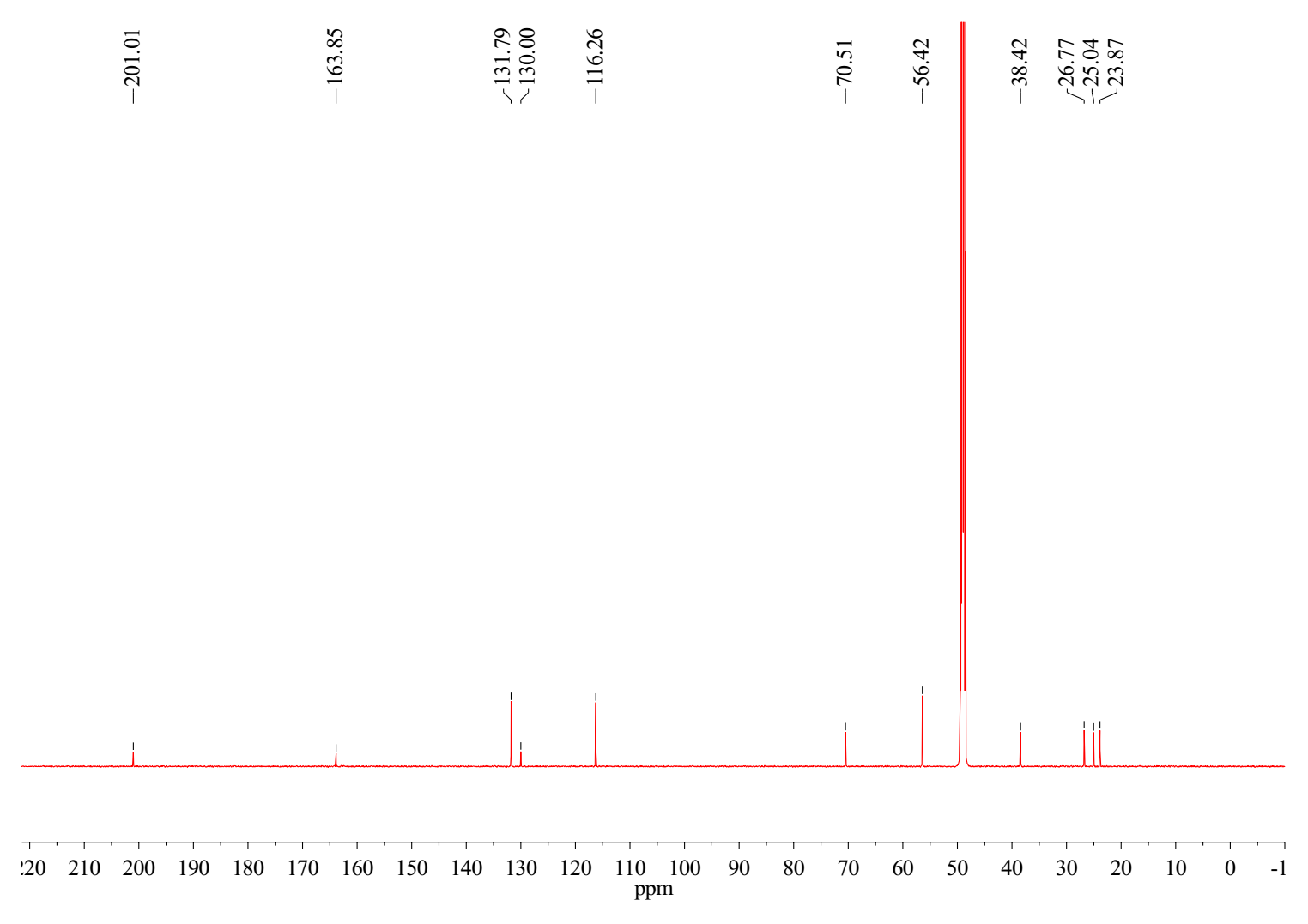


# Fig. S13. HSQC spectrum of compound 3 (in CD_3_OD, 600 MHz)


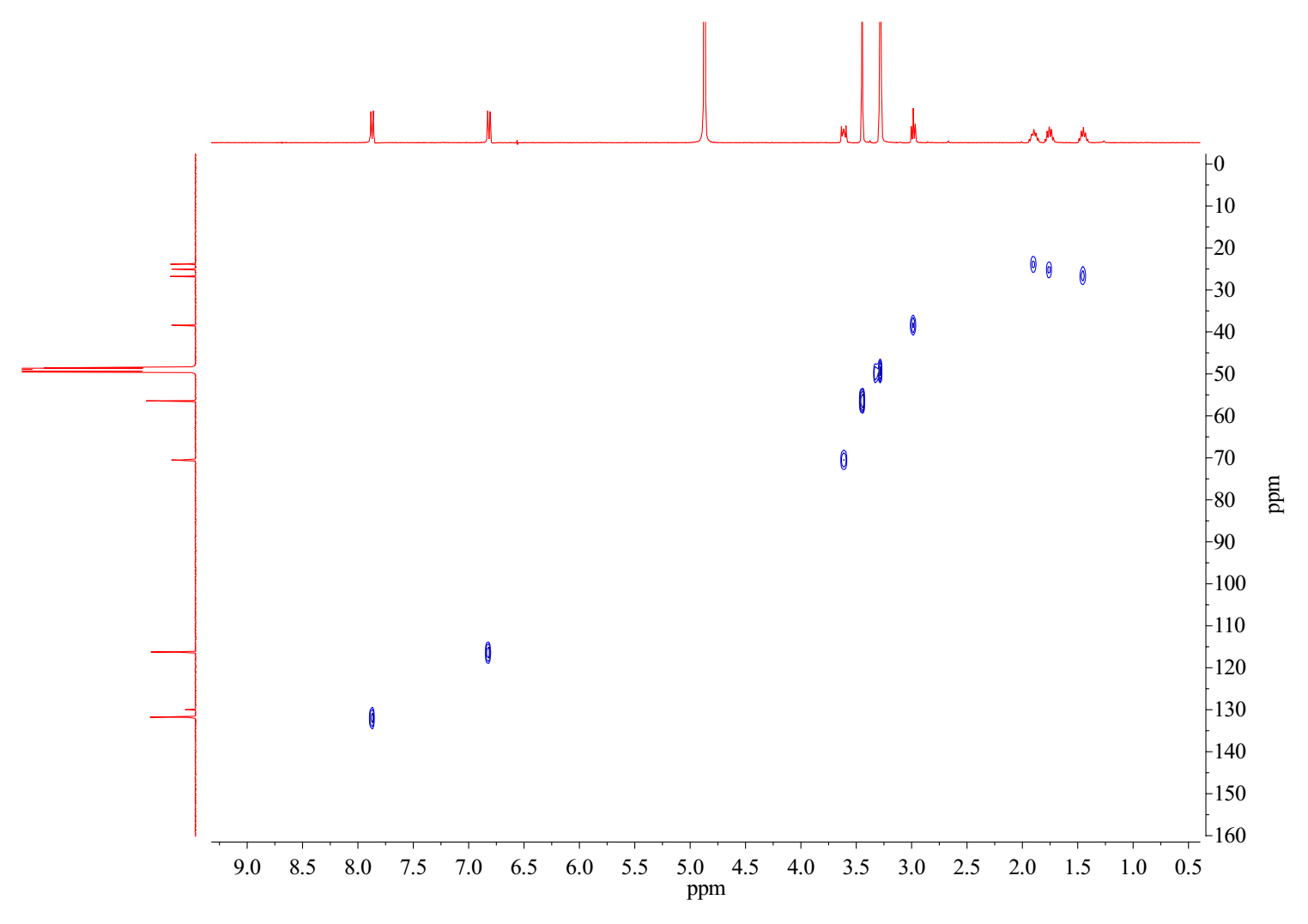


# Fig. S14. HMBC spectrum of compound 3 (in CD_3_OD, 600 MHz)


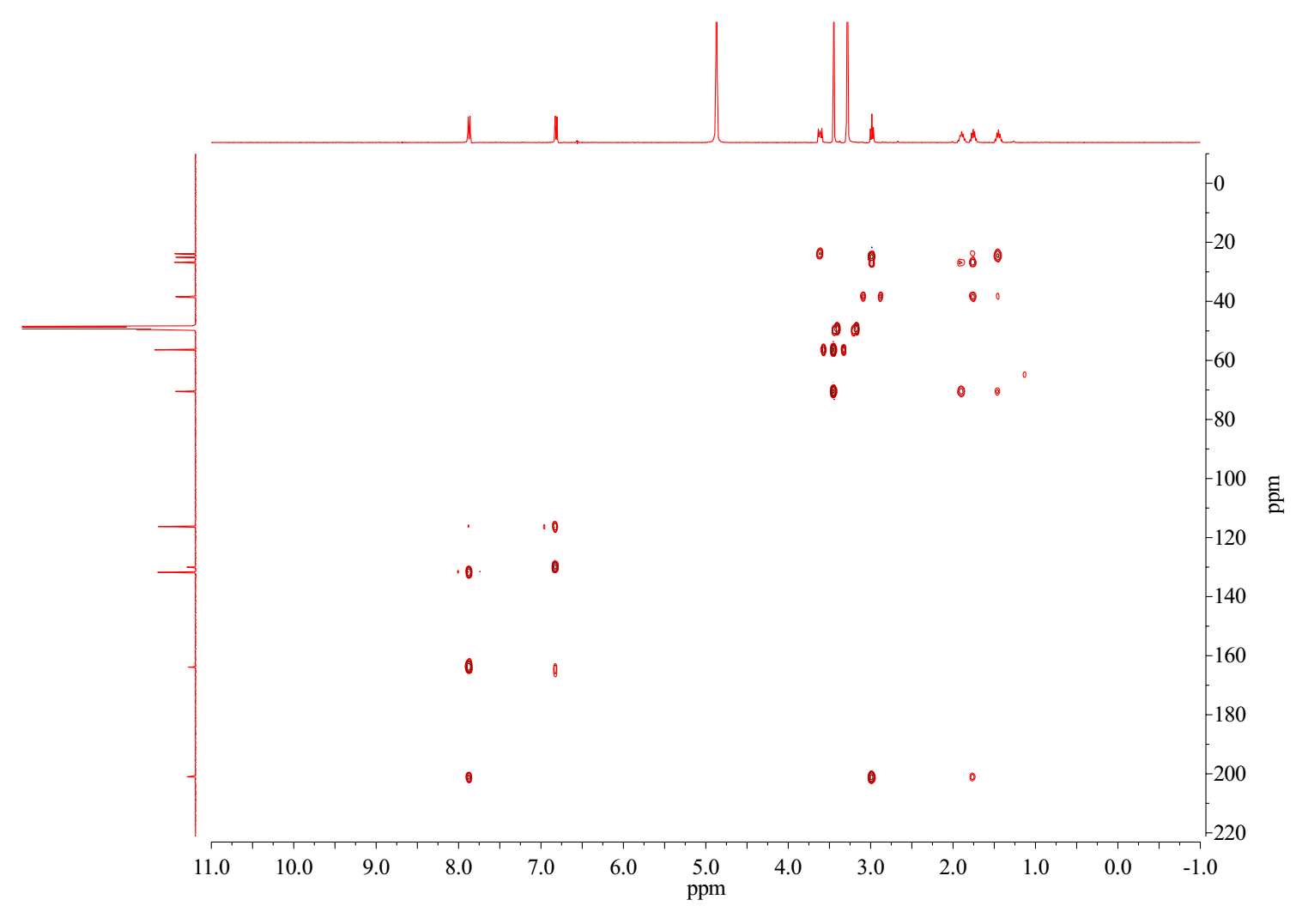


# Fig. S15. HRESIMS spectrum of compound 3


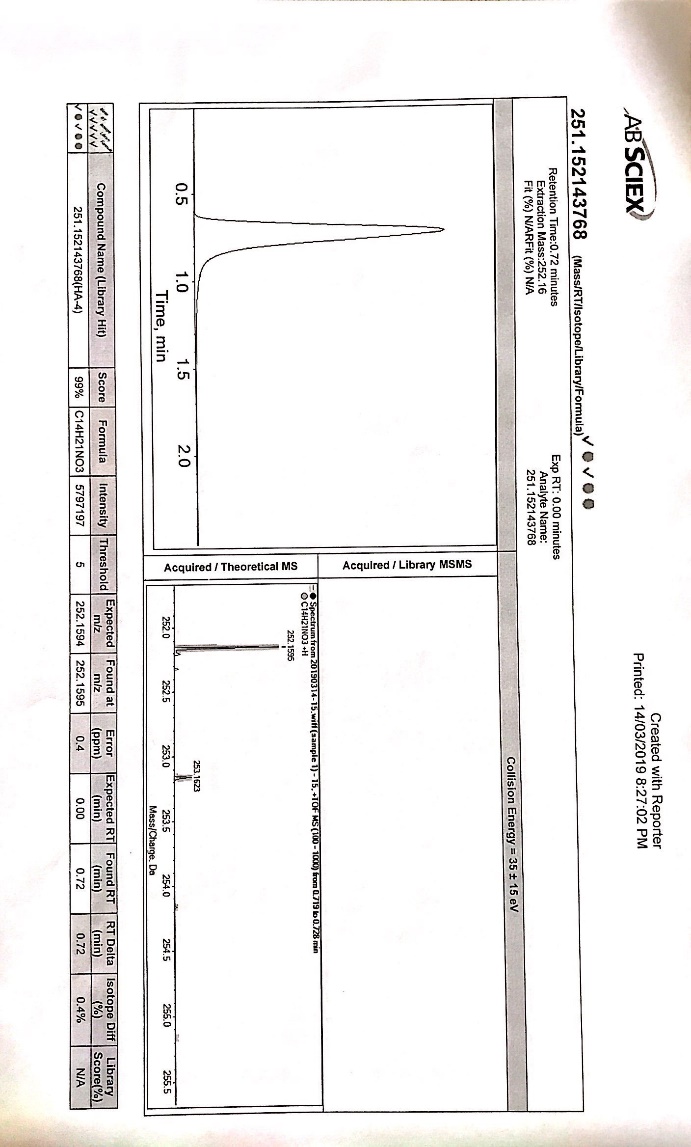


# Fig. S16. ^1^H NMR spectrum of compound 4 (in CD_3_OD, 400 MHz)


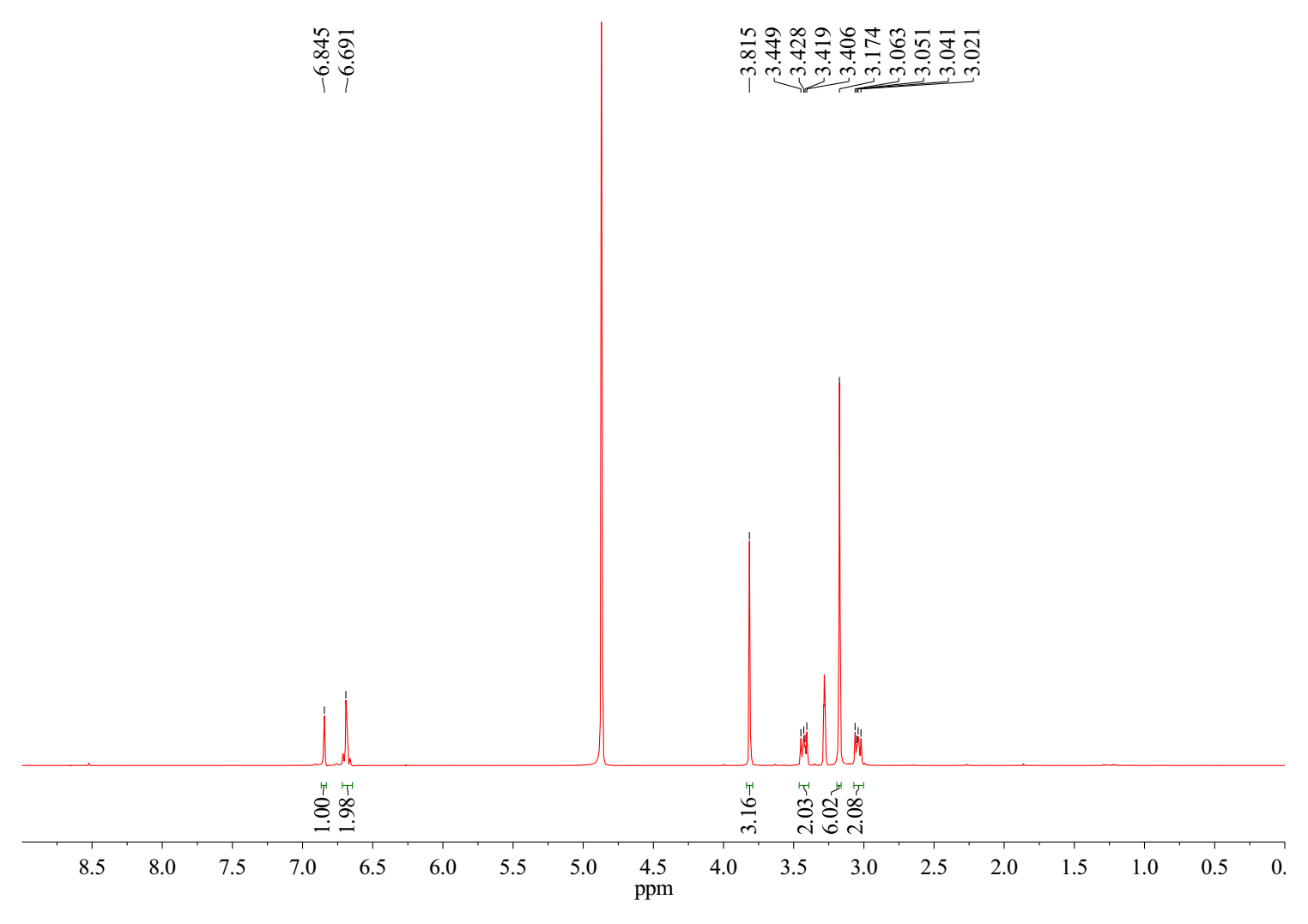


# Fig. S17. ^13^C NMR and DEPT spectra of compound 4 (in CD_3_OD, 150 MHz)


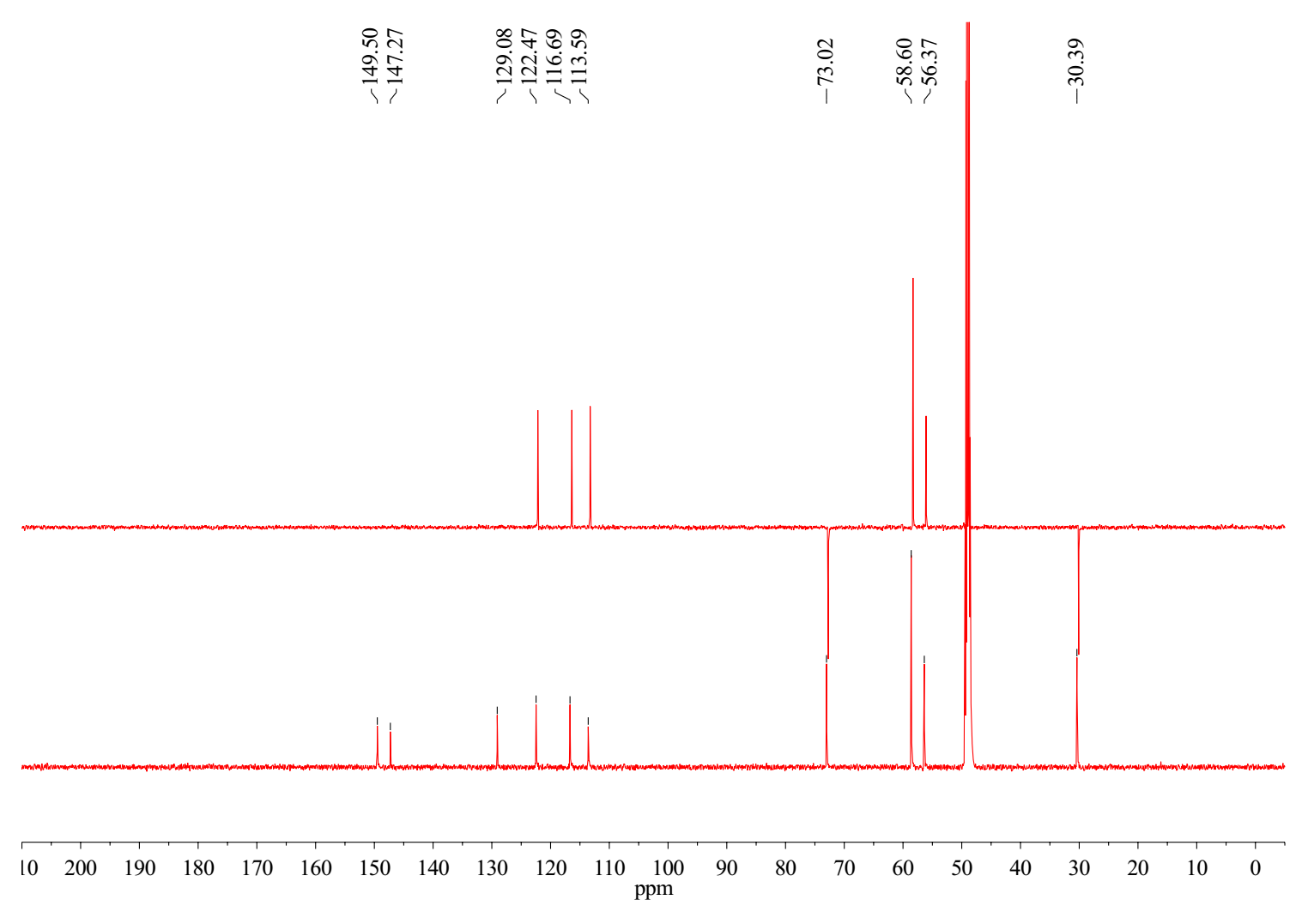


# Fig. S18. HSQC spectrum of compound 4 (in CD_3_OD, 600 MHz)


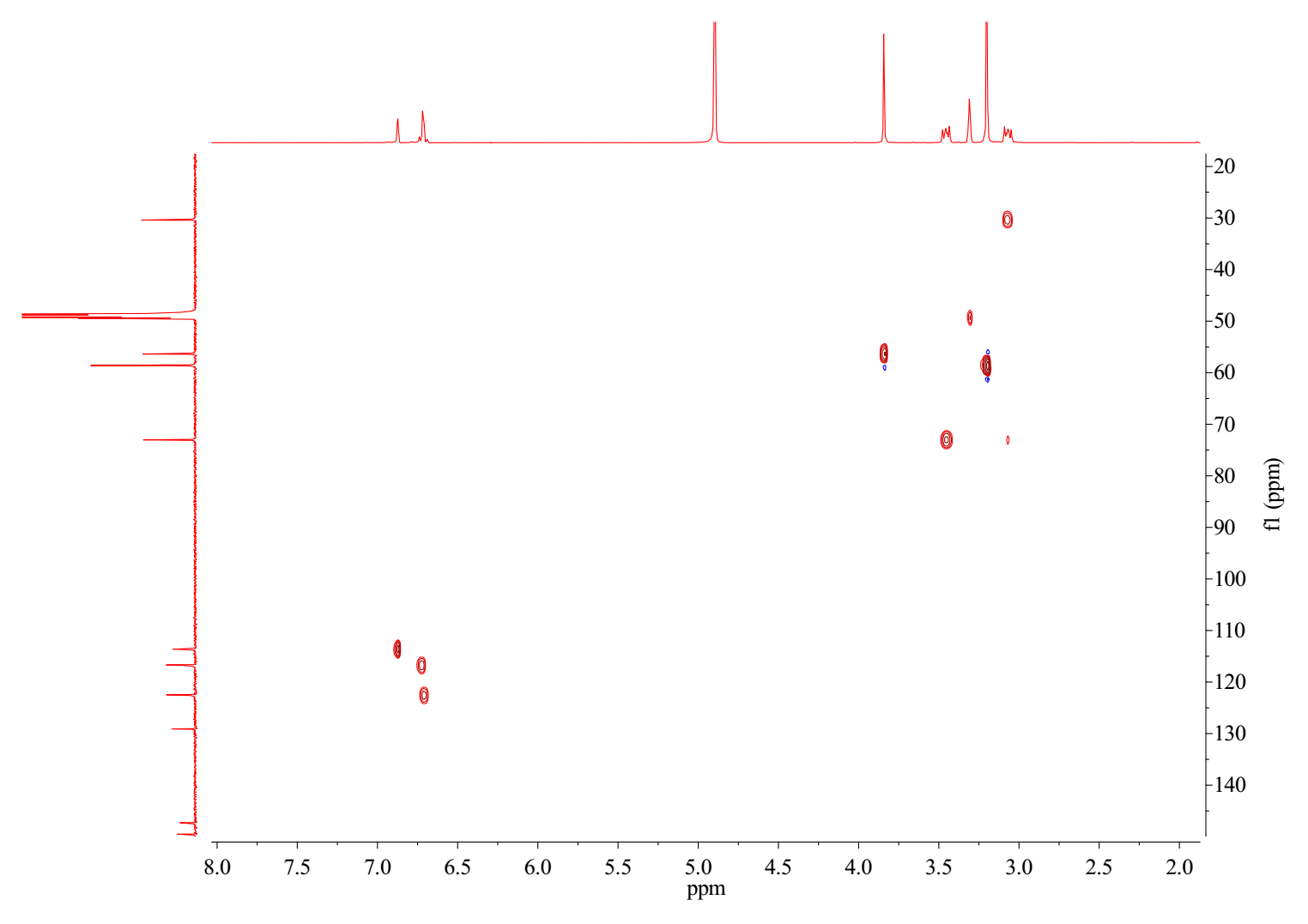


# Fig. S19. HMBC spectrum of compound 4 (in CD_3_OD, 600 MHz)


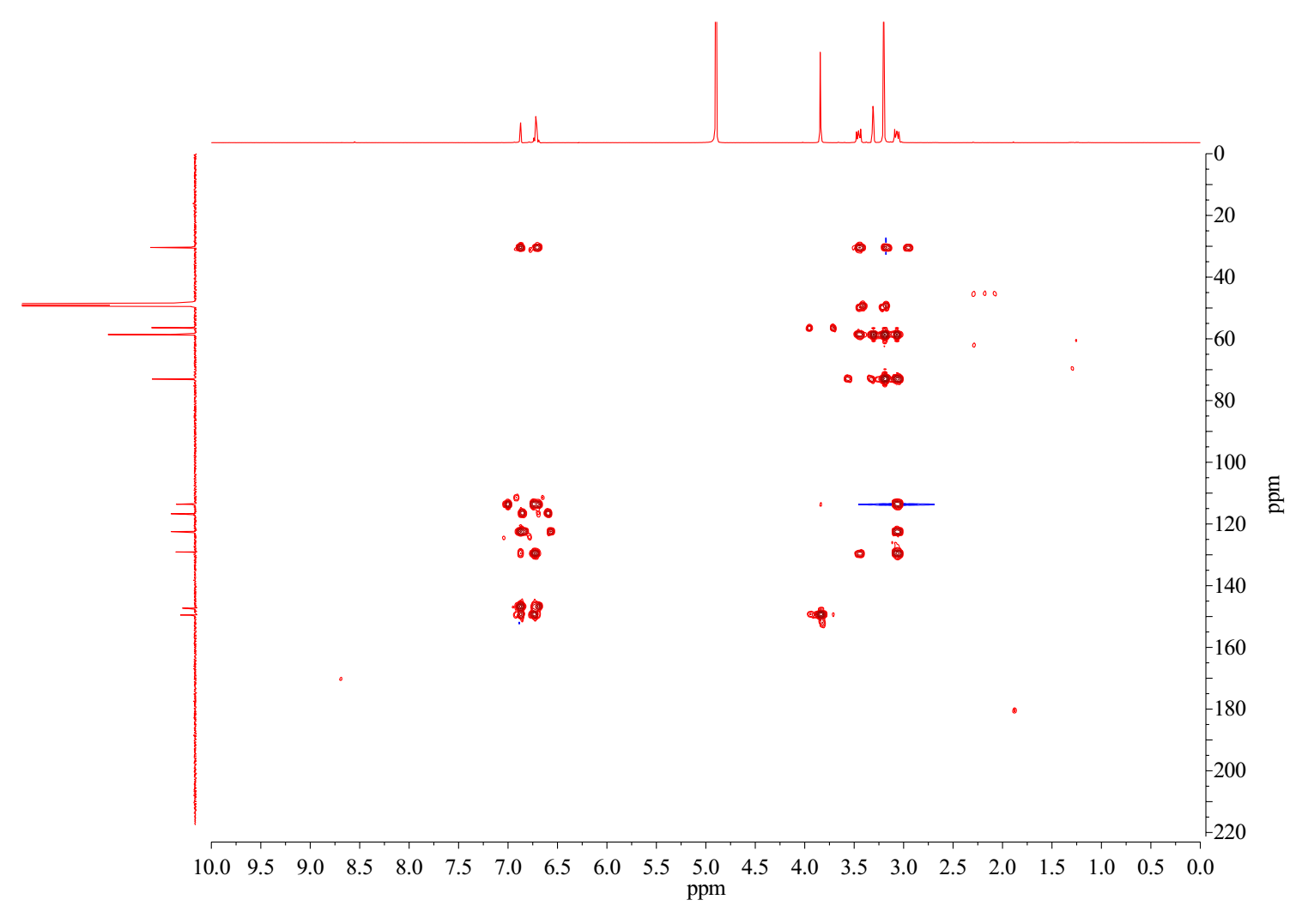


# Fig. S20. HRESIMS spectrum of compound 4


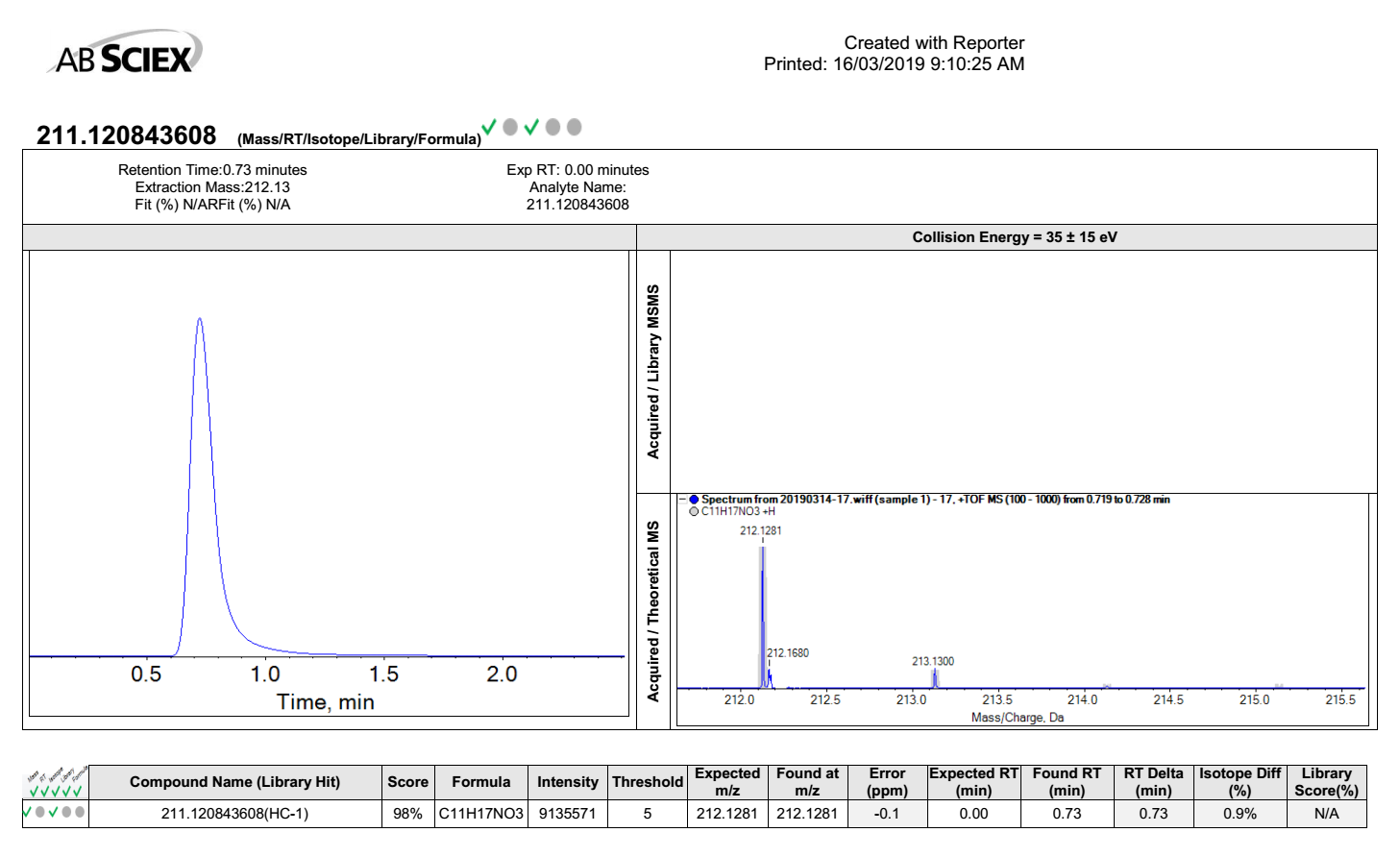

Supplement: Supplementary file 1 — Electronic supplementary material 1 (DOCX 6474 kb) [file 13659_2020_233_MOESM1_ESM.docx]
